# 

**Published:** 1877-09-20

**Authors:** 


☞All communications relating to the business of THE RECORD, for the year 1877, must be addressed to

DR. R. C. WORD,
Business Manger Southern Med. Rec.,
Atlanta, Ga.

☞Brief and practical communications are solicited on all subjects pertaining to medicine. also reports of cases in practice.

☞Send money by check, postal order or registered letter.

☞Write your name, post-office, county and State plainly.

### SPECIAL NOTICE.

Parties who receive this number as a specimen copy, are requested to subscribe. We are plain and cheap, but aim to be practical and useful. Try our journal, and see if it does not contain more practical items in less space, and at less cost, than any other journal in this country. May subscribe for six months, though it is best to order the back numbers, and have your volume complete.

☞Dr. E. will please excuse us for not publishing his article on "Medical Education and Medical Colleges." We fully endorse his views in the main, and but for a few very strong personal criticisms, we would have published it with pleasure. We are always willing to discuss principles, but wish to avoid personalities as far as possible, our object being to accomplish good. Our readers will remember that the editors of THE RECORD have never believed that a charter, with a building, and even a full corps of distinguished gentlemen as nominal trustees, is all that is necessary to constitute a first-class medical college. We have always believed that teachers of talent and character were equally necessary. Brains, apparatus and appliances can establish a good institution almost anywhere. Without these, it cannot be done, even in greater places than "the hub of Georgia."

### COLABORATORS AND CONTRIBUTORS.

L. B. Anderson, M.D., Va.
Swan M. Burnett, M.D., D. C
J. M. Bristow M.D., Texas
J. W. Booth, M.D., N C.
W. F. Barr, M.D., Va.
B. W. Corn, M.D., Texas
John Castor, M.D., Iowa.
E. T. Easley. A. M., M.D., Ark
A. H. Goelet, M.D., N. Y.
E. C. Gehrung, M.D., Mo.
G. D. Hodge, M.D., Ark.
S. M. Hogan, M.D., Ala.
A. McLane Hamilton, M.D.,NY
C. C. Vanderbeck, M.D., Pa.
Z. B. Herndon, M.D., Va.
Lindsay Johnson, M.D., Ga.
A. R. Kilpatrick, M.D., Tex.
R. Kells, M.D , Miss.
W. L. Lipscomb, M.D., Miss.
J. M. Lewis, M.D., Miss.
G. A. Dyer, M.D., Ind.
C H Lathrop, M.D., Iowa.
A A Lyon, M.D., La
M Lewis, M.D., Tenn
G M Rivers, M.D , S C
A S ayne, M.D., Va.
A. M. Whitehead, M.D., O.

